# Impact of climate change on allergic diseases in Germany

**DOI:** 10.25646/11654

**Published:** 2023-09-06

**Authors:** Karl-Christian Bergmann, Randolf Brehler, Christina Endler, Conny Höflich, Sabine Kespohl, Maria Plaza, Monika Raulf, Marie Standl, Roma Thamm, Claudia Traidl-Hoffmann, Barbora Werchan

**Affiliations:** 1 Charité – Universitätsmedizin Berlin Institute of Allergology Berlin, Germany; 2 Fraunhofer Institute for Translational Medicine and Pharmacology ITMP Immunology and Allergology Berlin, Germany; 3 University Hospital Münster, Department of Dermatology, Outpatient Clinic for Allergology, Occupational Dermatology and Environmental Medicine Münster, Germany; 4 German Meteorological Service Research Centre Human Biometeorology Freiburg, Germany; 5 German Environment Agency Section II 1.5 Environmental medicine and health effects assessment Berlin, Germany; 6 Institute for Prevention and Occupational Medicine of the German Social Accident Insurance, Institute of the Ruhr-Universität Bochum (IPA), Competence Centre Allergology/Immunology Bochum, Germany; 7 University Hospital Augsburg, Medical Faculty, Environmental Medicine Augsburg, Germany; 8 Helmholtz Zentrum München, German Research Center for Environmental Health, Neuherberg, Germany; 9 Robert Koch Institute Department of Epidemiology and Health Monitoring Berlin, Germany; 10 German Pollen Information Service Foundation Berlin, Germany

**Keywords:** CLIMATE CHANGE, ALLERGY, POLLEN, OCCUPATION, INHALANT ALLERGENS, AIR POLLUTANTS, MONITORING

## Abstract

**Background:**

Allergic diseases, especially inhalation allergies, have reached epidemic levels and environmental factors play an important role in their development. Climate change influences the occurrence, frequency, and severity of allergic diseases.

**Methods:**

The contents of this article were selected by the authors and developed section by section according to their expertise and the current state of knowledge. The sections were then discussed and agreed upon amongst all authors.

**Results:**

The article highlights direct and indirect effects of climate change on allergies. It goes into detail about the connections between climate change and (new) pollen allergens as well as (new) occupational inhalation allergens, explains the effects of climate change on the clinical picture of atopic dermatitis, discusses the connections between air pollutants and allergies, and provides information about the phenomenon of thunderstorm asthma.

**Conclusions:**

There is a need for action in the field of pollen and fungal spore monitoring, allergy and sensitisation monitoring, urban planning from an allergological perspective, and changes in the working environment, among others.

## 1. Allergies in times of climate change

Allergic diseases, especially inhalation allergies, have reached epidemic levels and environmental factors play an important role in their development. Climate change is accompanied by large-scale changes in our environment and thus influences the occurrence, frequency, and severity of allergic diseases.

This article highlights direct and indirect effects of climate change on allergies. The content of the paper was selected by the authors and developed section by section according to their expertise and the current state of knowledge. The sections were then discussed and agreed upon by all authors.

The article begins with definitions of terms and frequencies of allergic diseases and goes into detail on pollen as the main trigger of allergic respiratory diseases. Subsequently, connections between climate change and (new) pollen allergens as well as (new) occupational inhalation allergens are examined in more detail and effects of climate change on the clinical picture of atopic dermatitis are described, connections between air pollutants and allergies are explained, and information is provided about the phenomenon of thunderstorm asthma.

Recommendations on the topics of pollen and fungal spore monitoring, allergy and sensitisation monitoring, urban planning under allergological aspects, and changes in the working environment conclude the article.

### 1.1 Definitions of terms: Allergy, sensitisation, atopy

An allergy is an exaggerated response of the immune system to a normally harmless substance from the environment. Depending on the way the immune system reacts to this substance, the allergen, four types of allergy are distinguished, of which type I, also known as immediate-type, and type IV (contact hypersensitivity) are the most common (Table 1). Classic type I allergies are allergic rhinitis/rhinoconjunctivitis, also known as hay fever, and allergic bronchial asthma. The classic representative of a type IV allergy is allergic contact dermatitis. Why the immune system reacts allergically, i.e. in an exaggerated manner, to some substances from the environment has not been conclusively clarified.

In the context of allergies, we speak of type I sensitisation if allergen-specific immunoglobulin E (IgE) antibodies can be detected in the blood and/or if the skin test (prick or intradermal test) is positive. After sensitisation, renewed exposure to the allergen in allergic people leads to the release of mediators eliciting the allergic symptoms.

Atopy (atopía, Greek = placelessness) is a familial predisposition to develop allergic diseases (especially of the immediate-type/type I) based on an immunological hyper-sensitivity of the skin and mucous membranes to allergens, which is accompanied by an increased production of IgE antibodies and the formation of allergen-specific IgE antibodies. Atopics are thus a subgroup of people with allergies and often show some typical clinical manifestations. These include a double fold on the lower eyelid (Dennie-Morgan fold), dark skin around the eyes, thinning of the lateral eyebrows (Hertoghe’s sign), predominantly dry and itchy skin, and a dry scalp.

### 1.2 Frequencies of allergic diseases and sensitisations

Worldwide, the prevalence of allergic diseases has increased strongly over recent decades and is currently stagnating at a high level. It is estimated that a total of 20 to 30 million people in Germany are affected by allergies [2], and the age of onset tends to decrease [3]. The increase in allergic diseases has been accompanied by concurrent changes in lifestyle and environment. Many factors associated with these changes were linked to the increased occurrence of allergic diseases, as illustrated, for example, by comparisons between East Germany and West Germany or by farm studies [4].

#### Cross-sectional population data

Population-representative data on the epidemiology of allergic diseases in Germany are provided by the ongoing health monitoring at the Robert Koch Institute [5]. For adults, the most recent data are from the nationwide survey ‘German Health Update’ (GEDA 2019/2020-EHIS), which was conducted between April 2019 and September 2020. This survey includes the questionnaire of the European Health Interview Survey (EHIS), which takes place every five years. Based on respondents' self-reports, 8% of adults currently suffer from bronchial asthma (including allergic asthma), i.e. in the last 12 months before the survey. Almost one third of adults (31%) reported that they were currently affected by an allergy. Allergies asked about in the GEDA 2019/2020-EHIS were hay fever, allergic reactions of the eyes or skin, food allergies, or other allergies (except allergic asthma). Overall, women reported being affected by allergic diseases more frequently than men [6].

A detailed survey of the frequency of physician-diagnosed allergic diseases was last carried out in the nationwide study ‘German Health Interview and Examination Survey for Adults’ (DEGS) from 2008 to 2011. At that time, around 16% of adults stated that they had received a medical diagnosis of hay fever (allergic rhinitis). Allergic rhinitis is thus the most common allergic disease. The lifetime prevalence of bronchial asthma was around 9% (however, not all types of asthma have an allergic genesis). Allergic contact dermatitis, which had been diagnosed by a doctor in about one in eleven adults (9%) at some time in their lives, was similarly frequent to bronchial asthma. Less prevalent were food allergies (5%), atopic dermatitis (4%), and insect venom allergy (3%). With the exception of atopic dermatitis, women were more often affected by allergic diseases than men [7].

Sensitisation is even more common than allergic diseases. The DEGS study showed that allergen-specific IgE antibodies against environmental allergens were detectable in the serum of 50% of adults in Germany. For example, 34% of adults were sensitised to a mixture of timothy, rye, birch, mugwort, cat, dog, dust mite, and *Cladosporium herbarum* (sx1 allergen mixture), 26% to food allergens (with only 2% being sensitised exclusively to food allergens), and 19% each to grass and tree pollen. 11% were sensitised to herb pollen [8].

For children and adolescents, population-based prevalence of allergic diseases and sensitisation were most recently derived on the basis of the second follow-up survey of the ‘German Health Interview and Examination Survey for Children and Adolescents’ (KiGGS Wave 2; data collection 2014 to 2017). According to this study, 11% of all children and adolescents aged 0 to 17 years had received a medical diagnosis of hay fever at some point in their lives, and the figure for bronchial asthma was 6%. Boys were affected more often than girls. The lifetime prevalence of atopic dermatitis was 13%, with girls being affected more frequently than boys. In both girls and boys, 3% had already been diagnosed with allergic contact dermatitis by a doctor at some point in their lives [9].

37% of children and adolescents in Germany were sensitised to the sx1 allergen mixture consisting of the above-mentioned eight inhalation allergens. The prevalence of sensitisation to timothy and rye pollen, birch pollen, and dust mites was between 14 and 23%, and the prevalence of sensitisation to most of the animal and food allergens tested was between 5 and 11%. Boys were generally more frequently sensitised than girls.

A factor analysis regarding sensitisation patterns identified seven sensitisation groups for girls and boys alike, namely timothy/rye pollen, birch pollen/apple, food/mugwort pollen, dust mites, animals, cow’s milk/egg white and mould [9, 10].

#### Population-based longitudinal data

Studies on the development on an individual level (longitudinal) within the KiGGS cohort showed that every fifth girl (21%) and every third boy (29%) newly developed a sensitisation to at least one of the sx1 allergens in the course of ten years of life (cumulative 10-year incidence). Additionally, it was shown that once sensitisation had been detected, it persisted for the most part. Only in 11% of the affected girls and 6% of the affected boys was sx1 sensitisation no longer detectable (remission) ten years later [11]. Longitudinal results in adults indicate that the observed increase in the prevalence of sx1 sensitisation is most likely a cohort effect, due to a higher prevalence in younger cohorts [12].

### 1.3 Pollen as an allergy trigger

The most common trigger of allergic respiratory diseases are pollen or, more specifically, the allergens they contain.

Pollen consists of pollen grains, which can range in diameter from less than 10 μm (e.g. woodland forget-me-not pollen) to more than 100 μm (e.g. white fir pollen) [13].

Pollen is part of plant reproduction. The transfer of pollen grains from the male anther to the female stigma is called pollination. There are two basic types of pollination: in autogamy (self-pollination), the pollen is transferred to the stigma of the same flower or to the stigma of another flower of the same plant (geitonogamy). In allogamy (cross-pollination), the pollen from one plant is transferred to the flower of another plant. Pollination occurs through transport vectors such as water (hydrogamy), animals (zoogamy) or wind (anemogamy). The most common way of pollen transfer is zoogamy, in Germany specifically insect pollination (entomogamy, e.g. in dandelions or apple trees).

However, from an allergological point of view, anemophilous plants, i.e. plants with characteristics that favour the transmissibility of pollen by wind, play the most important role in Germany; most allergy-relevant pollen taxa belong to this group. The usually large amount of pollen of anemophilous plants in the air leads to an increased human exposure to the pollen of these plants, which also increases the possibility of developing sensitisation and allergic symptoms. Typical representatives of anemophilous plants with allergy-relevant pollen are hazel, alder, birch, oak, grasses, and mugwort. However, pollen of entomophilous plants can also become airborne in quantities sufficient to trigger sensitisation or allergy in Germany (e.g. tree of heaven pollen [14]).

## 2. Climate change and allergies: Direct and indirect effects

As mentioned in the introductory article of this status report [15], the mean air temperature in Germany has increased by about 1.6°C since the beginning of nationwide weather records in 1881. By the end of the century (2071–2100), an increase in the annual mean temperature of at least 1°C to more than 4°C is expected in Germany, depending on the emissions scenario, with the strongest warming in the Alps and the Alpine foothills. Overall, the warming is expected to be similar in the different seasons, with the exception of spring, where model calculations show a slightly lower warming [16, 17]. Precipitation in Germany has increased by 8% in the annual sum since 1881. While the greatest increase is in winter, followed by spring and autumn, summer precipitation has decreased slightly. By the end of this century, a further 8% increase in annual precipitation is expected compared to the period 1971 to 2000. The largest increases of up to 17% will occur in winter, while a decrease in precipitation is expected in summer. It should be noted that modelled changes below 10% cannot be distinguished from natural climate variability [17].

In addition to temperature and precipitation, evaporation and soil moisture also play a role in plant development. Evaporation usually increases with higher temperatures. In addition, if precipitation changes only slightly, soils can be expected to dry out more quickly during the vegetation period if land use does not change. The number of days with low soil moisture has already increased significantly since 1961 and will continue to increase [17, 18]. Consequently, the water availability for plants within the vegetation period decreases and dryness and drought can be expected to increase. From 1971 to 2000, the average number of months of drought in Germany was about two months per year. A warming of 3°C would double the drought duration [19]. At the same time, a rise in temperature leads to an increase in the potential for extreme precipitation events, which in turn can lead to increased flooding [20]. With the increase in extreme events, the potential for so-called thunderstorm asthma also increases (Info box).

### 2.1 Climate change and (new) pollen allergens

#### Changes in the phenological development of pollen-producing plants

**Figure 1:**
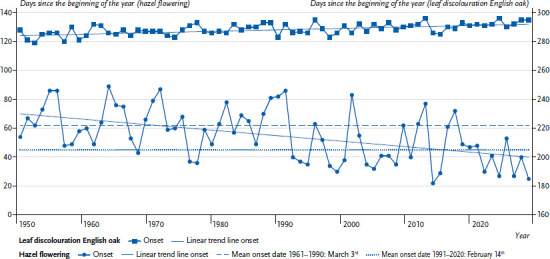
Start of flowering for hazel and leaf discolouration of English oak since 1951 as indicators for the start and end of the vegetation period Source: Deutscher Wetterdienst [26] (original language German)

Plant development and thus the pollen season depend significantly on the interaction of temperature and precipitation. The last decades of the 20^th^ century saw a significant shift in the phenological seasons, i.e. the onset of different developmental stages of plants (from flowering to leaf fall) [17], which also resulted in a shift of the pollen season. In hazel, for example, the onset of flowering has shifted forward by about one month since 1951, while the beginning of leaf discolouration of English oak (late autumn), which is used here as an indicator for the end of the vegetation period, has occurred only slightly later (Figure 1). The end of the growing season is generally controlled less by temperature than by day length. As a result, the onset of late autumn and the onset of winter remain relatively constant, so that the earlier onset of spring lengthens the growing season [17]. A similar trend to that of hazel can also be seen with black alder, a widespread alder species in Germany. In addition, the non-native Spaeth’s alder (Alnus × spaethii) also starts the alder pollen season earlier. In very mild winters and at favoured locations, hazel and Spaeth’s alder can even begin to flower as early as November [24, 25]. In some years, the last grass and nettle pollen also flew in November [25].

**Info box Extreme weather and asthma**

In the course of climate change and increasing extreme weather events, the phenomenon of thunderstorm asthma could also become more significant in Germany. Severe asthma attacks can occur during thunderstorms, especially in people with hay fever and allergic asthma. Asthma has also been observed during thunderstorms in people who only suffer from hay fever. The phenomenon of thunderstorm asthma has occurred relatively rarely so far: worldwide, about 30 such events have been recorded since 1983, mainly in Australia and England. The exact mechanisms of thunderstorm asthma are not yet fully understood [21–23].

Essential features of thunderstorm asthma are:

► Occurrence mainly in late spring and summer during special weather events such as thunderstorms or convergence lines
► (Unusually) high concentration of aeroallergens in the air, sometimes even several days before the weather event (aeroallergens in connection with thunderstorm asthma are mainly grass pollen, but also tree and herb pollen as well as fungal spores; weather changes such as precipitation, increase in humidity and lightning activity can fragment pollen, creating smaller, easily respirable particles to which allergens are bound and which are transported to the ground by strong downdrafts)
► Onset of symptoms often during the first 20 to 30 minutes of the weather event (increased risk of severe symptoms such as acute asthma attacks, increased risk of emergency room visits)

Phenology and pollen season are not necessarily synchronous. For example, pollen from climatically favoured areas, where flowering has already started, can be transported over long distances to areas where flowering has not yet started, thus causing the pollen season to start earlier there as well.

It is likely that ongoing climate change will lead to an even earlier start of the pollen season, even though plants in temperate latitudes often require a certain degree of cold exposure (chilling) during the cold season for flower induction, and chilling may no longer be adequately fulfilled due to the rise in temperature. Ettinger et al. [27], for example, found that a winter warming of more than 4°C (mean temperature) can lead to a delay in spring phenology due to reduced cold exposure.

#### Influence of climate change on plant productivity

Due to increasing CO_2_ concentrations, an increase in the amount of pollen is to be expected, as shown, for example, by experiments on ragweed [28, 29] and timothy [30]. High pollen concentrations also occur in so-called mast years. In these years, increased seed production occurs in certain tree species. Mast years are subject to a specific cycle; for beech, for example, mast years occur approximately every three to six years and for oak every six to twelve years [31]. Even if the pollen of beech and oak (beech family) has low allergy potential, allergen exposure can be increased by cross-reactivity to pollen of botanically related species like birch, alder, hazel (birch family). In addition, mast years have become more frequent in recent years, which is associated with climate change [32, 33]. If the frequency of mast years increases, trees could in turn become more susceptible to, for example, pests and drought due to the increased energy demand. If trees in temperate latitudes are exposed to a persistent lack of water, this has a negative effect on their development, for example, through a decline in pollen production [34–36]. Drought or prolonged periods of drought will occur more frequently in the future, as recent years have shown [19]. The alternation of weather extremes (drought and flooding due to heavy rainfall events) can lead to drought stress and waterlogging, which not every tree can handle. For example, a creeping deterioration of the general condition of urban birch trees can be observed [37].

Due to the influence of climate change on plant development, there are changes in the exposure of the population to allergenic pollen. These concern: (a) the timing of pollen flight; (b) pollen concentrations; (c) the pollen spectrum; and (d) the allergenicity of pollen, among other things in connection with changes in air quality, for details see Section 2.4 Air pollutants.

#### Changes in the timing of pollen occurrence

Using the symptom-oriented definition of a pollen season by the European Academy of Allergy and Clinical Immunology [38], the strongest changes can be observed in the birch pollen group (e.g. hazel, alder, birch, beech, oak). The seasonal onset of this group has occurred about two to three weeks earlier in recent decades, but also ended earlier [39–41]. The earlier start of the birch pollen flight has also been recorded in the national pollen calendars, which have been published for decades by the German Pollen Information Service Foundation (Stiftung Deutscher Pollen-informationsdienst, PID) [25]. Similar observations have been made for beech pollen at two PID monitoring sites located several hundred kilometres apart and at different altitudes [42].

The flight of grass pollen has changed less, tending to start earlier. In some countries (United Kingdom, Spain, Portugal) a prolongation of the flight of grass pollen has been observed [43]. As a result of warmer autumn months, a prolonged flight of mugwort and ragweed pollen can also be expected; based on measurements between 2011 and 2016, the pollen calendar 4.0 indicates a possible occurrence of mugwort and ragweed pollen from June to the end of October/beginning of November [25].

Overall, the earlier start of the tree pollen season and the extension of the herb pollen season into autumn results in a spread of the pollen season for allergenic plant species. This prolongs the symptom period for those who are allergic to tree pollen as well as grass and herb pollen. Since polysensitised people generally have a higher risk of developing more severe symptoms and bronchial asthma [44], the extended exposure period poses a particular risk for this group of people.

#### Changes in pollen concentrations

**Figure 2:**
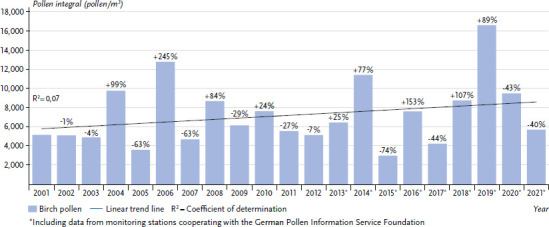
Overview of the mean number of birch pollen measured per year at various monitoring stations in Germany with trend line. Percentages show the respective change compared to the previous year. Source: German Pollen Information Service Foundation [46] (original language German)

The annual sums of the allergenic pollen relevant in Germany have been subject to constant changes over the last two or three decades. For example, in the case of birch, a year with a high pollen release was often followed by a year with a lower release. However, the average annual pollen sum for birch has tended to increase (Figure 2). For the PID monitoring site in Munich, the number of days with high birch pollen concentrations (≥ 100 pollen/m^3^) has also increased significantly [45].

The only previously published data on the course of the annual pollen sum of 23 pollen types from 97 measurement sites in Europe over recent decades showed significant increases for ten of them, including alder, birch, hazel, ash, plane, oak, and cypress. In contrast, the annual pollen sum of mugwort showed a significant decrease [47].

However, the severity of allergic symptoms does not depend solely on the pollen concentration as the allergen content of the pollen is also important. A grass pollen grain contains < 1 to 9 picograms (pg) Phl p 5 (major allergen of grass pollen), the average value is 2.3 pg Phl p 5. However, grass pollen does not release Phl p 5 continuously, so that patients can be symptom-free despite pollen flight depending on climatic conditions. The concentration of free allergens increases with humidity [48]. The symptoms of people with allergies thus depend not only on the extent of the pollen count, but also on climatic conditions [49].

#### Changes in the spectrum of allergenic pollen

With climate change progressing, the spectrum of allergenic pollen in Germany will very likely continue to change:

(A) The allergological significance of pollen from some free-growing but non-native plant species will increase.

(B) New pollen allergens will occur.

(C) The allergological significance of pollen of some native plant species may change.

Scenario A is discussed in more detail below. Scenario B is exemplified by olive tree pollen [50, 51], scenario C by birch tree pollen [52].

##### Scenario A, example 1: Ragweed pollen

The common ragweed (*Ambrosia artemisiifolia*) is native to North America and produces large amounts of pollen with high sensitisation and allergy potential [53, 54]. In the United States, as many people are sensitised to ragweed as to grasses [55]. The plant probably reached Europe via cereals or clover seed and is now widespread mainly in Ukraine, Hungary, Italy (Po Valley), and France (Rhone Valley). In Germany, it was found growing wild as early as 1860 and for a long time was considered impermanent and rare. However, for several years it has continued to spread [53, 54]. In addition to human-aided entry, current changes in climate seem to be promoting the growth of the plant and its pollen and allergen production [56].

**Figure 3:**
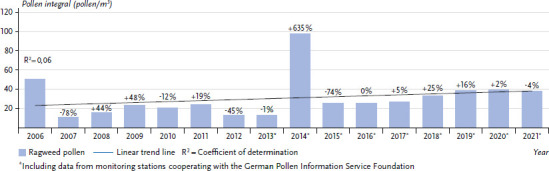
Overview of the mean number of ragweed pollen measured per year at various monitoring stations in Germany with trend line. Percentages show the respective change compared to the previous year. Source: German Pollen Information Service Foundation [46] (original language German)

In 2006 and 2014, considerable amounts of ragweed pollen were detectable in Germany, having probably entered via long-distance transport from Hungary (Figure 3). Due to existing cross-reactivities to the native mugwort, clinical complaints in patients allergic to mugwort could have occurred despite the one-off event. However, there are no data on this.

Compared with the long-distance transport events of 2006 and 2014 and with the annual average for Germany as a whole, significantly higher pollen concentrations have been detectable in the region around Drebkau in south-eastern Brandenburg for several years, resulting from the establishment of the plant in this region (Table 2).

The clinical relevance of this exposure, which has existed for years, has not yet been comprehensively investigated. However, data from Italy suggest that primary sensitisations to ragweed pollen in the region around Drebkau are now likely to be significantly higher than the national average [58].

For the years 2041 to 2060, Lake et al. [59] predict that clinically relevant concentrations of ragweed pollen will occur as early as July/August for areas in southern Germany based on different climate models, different plant dispersal models, and two representative concentration pathways (RCP) scenarios (RCP4.5 and RCP8.5). Furthermore, pollen concentrations in Germany will be at least twice as high in the main flowering season as in the comparative period 1985 to 2005. Pollen in clinically relevant amounts will also be detectable in the off-season [59]. With regard to population-related sensitisation to ragweed pollen, an increase from 0 to 10% in the period 1985 to 2005 to an increase of 15 to 25% in the period 2041 to 2060 is assumed for Germany [59].

##### Scenario A, example 2: Wall pellitory pollen

The wall pellitory (*Parietaria officinalis*) is widespread in central and southern Europe and is considered an archaeophyte in Germany (i.e. plant species permanently established before 1492). In southern European countries, patient-related sensitisation rates against wall pellitory are around 20%. In Germany, the percentage is below 10% (prick test data: [60], allergen-specific IgE data: [61]). With climate change progressing, wall pellitory plants could spread in Germany and subsequently lead to an increase in the number of patients with wall pellitory allergy.

##### Scenario A, example 3: Pollen of the tree of heaven

The tree of heaven (*Ailanthus altissima*) is present on all continents except Antarctica, but is only native to parts of Asia. In Germany, it is currently mainly found within urban heat islands. It is expected to spread beyond these heat islands as warming continues [62]. The tree is predominantly insect-pollinated, but its pollen can also be dispersed by wind and, beyond possible cross-reactivity, could also become allergenic in this country [62–64].

### 2.2 Climate change and (new) occupational inhalant allergens of animal, plant, and microbial origin

As a result of climate change, the conditions of employees in various workplaces will change in many ways. The agricultural and forestry sector, with more than 44 million jobs in the European Union (about 9.2% of the total workforce) and almost one million workers in Germany [65], is highly vulnerable to climate change. Studies point to strong regional differences in the spatial distribution of climate impacts. In the northern regions, for example, this is noticeable in the variability of crop yields and in an increase in pest infestations and diseases, which in turn can lead to health problems for the exposed employees.

#### Oak processionary moth

The oak processionary moth (*Thaumetopoea processionea*) belongs to the moth family Notodontidae. The stinging hairs of the caterpillar can cause both dermal (so-called caterpillar dermatitis) and respiratory complaints in humans. The pine processionary moth (*Thaumetopoea pityocampa*) and the northern pine processionary moth (*Thaumetopoea pinivora*) belong to the same subfamily. Their distribution has so far been described mainly in southern Europe, but is expected to also spread to Germany due to progressive climate change.

Processionary moths, mostly the oak processionary moth in Germany so far, are beneficiaries of climate change. They cause forestry damage in infested areas and pose a health hazard to humans, including landscapers and arborists. Fluctuating weather conditions can have a major impact on the development of this moth [66]. Very strong populations have been observed in the spring months during mild weather when conditions were good (little wind and precipitation, lots of sun) especially during moth flight and egg laying in the preceding late summer.

The third larval stage, in particular, poses the health hazard, as the caterpillars form stinging hairs that contain the protein thaumetopoein (nettle poison). The fine hairs break easily, can fly hundreds of metres with the wind and attach themselves to the skin of humans and animals via barbs. Direct contact with the stinging hairs of the oak processionary moth can cause mechanical-irritative, toxic and also allergic reactions, which can lead to skin irritation, eye irritation, fever, dizziness, and in individual cases even allergic shock. Breathing in the fine hairs can also cause respiratory problems such as bronchitis and asthma.

#### Hard ticks

Other beneficiaries of climate change include hard ticks, which prefer warmer air temperatures and high humidity. Previous warm summers and mild winters lead to early tick proliferation in spring. A tick species that is widespread throughout Europe is the castor bean tick (*Ixodes ricinus*). A more detailed overview of the influence of climate change on hard ticks and other vectors can be found as part of this status report in Beermann et al. [67].

The castor bean tick is spreading to more northerly and more highly elevated regions due to milder winters. It is not only significant as a vector of Lyme disease and tick-borne encephalitis, but can also induce sensitisation. A study in south-west Germany showed that forestry workers and hunters have a high prevalence of alpha-gal syndrome caused by tick bites. This can lead to an IgE-mediated (delayed) allergic reaction to red meat (e.g. beef, game) [68].

##### Cryptostroma corticale

Another health problem that could be amplified by climate change and particularly affects workers during logging, including wood processing, is the fungus *Cryptostroma corticale*. Since the early 2000s, the fungus has also appeared in Europe due to drought and warmer summers. Drought and hot spells have led to an increased outbreak of sooty bark disease in maple trees caused by *Cryptostroma corticale* in recent years. Infestation with *Cryptostroma corticale* discolours the wood and makes it unusable for further processing. On the one hand, this leads to considerable economic damage in the timber industry, and on the other hand, inhalation of the conidiospores can lead to health problems in exposed persons causing exogenous allergic alveolitis (type III allergy, see Table 1). This poses another occupational risk (co-)created by climate change [69].

#### Moulds

Increased humidity combined with higher temperatures and CO_2_ levels promotes fungal growth. Workers who carried out renovation work immediately after flood events were exposed to increased levels of mould [70]. In addition to spores, fragments of mycelial filaments (0.2 to 10 mm in length) are airborne allergen carriers that can occur in even greater quantities than spores. One can therefore be exposed to mould allergens through both spores and mycelial fragments. Mould exposures can cause various diseases of the upper and lower respiratory tract and the skin, for example allergic rhinitis or asthma.

#### Cannabis plants

Cannabis plants are also among the beneficiaries of climate change, as the plant thrives better under increased UV radiation exposure, which can be a result of climate change. In addition, cannabis and hemp plants are becoming increasingly important as raw materials with a growing range of products for fibre products, food and medicines. The increasing range of hemp-based products is leading to more and more employees working in this growing industry. Exposure to components of the cannabis plant in these workplaces is causing an increasing number of health problems, especially allergic complaints [71]. Sussman et al. [72] point out that the increase in cannabis use could lead to a scenario comparable to that caused by natural rubber latex exposure in the health sector in the 1980s and 1990s.

#### New allergens associated with food and feed production

In addition to the direct influences of climate change on allergen exposure in workplaces and thus often also on the health of employees, certain lifestyle changes are happening as a result of climate change (e.g. vegan diets, insects as a food source, fermented plant products as a substitute for meat). One consequence of these lifestyle changes is the increased use of enzymes in food production, which in turn can lead to sensitisation and allergic complaints [73].

Developments and processes that counteract climate change in the pursuit of sustainability can lead to new or modified products and thus different manufacturing processes and exposures in workplaces. This can lead to climate change having an indirect effect on the health of working people, one example being the exposure and sensitisation risk in the production of phytase as a feed ingredient of non-ruminants (poultry, pigs) [74]. Phytase can reduce the addition of inorganic phosphate and thus the wastewater load of excreted phosphate.

### 2.3 Atopic dermatitis and climate change

Atopic dermatitis (atopic eczema) is a chronic, itchy, inflammatory skin disease that is a considerable burden for patients [75].

It is triggered and exacerbated by environmental changes like those already occurring in Germany and Europe due to climate change, such as longer periods of heat, accumulation of tropical nights, and higher average temperatures. People suffering from atopic dermatitis should take measures to protect themselves from UV radiation and heat. Early warning systems can enable patients to better plan their daily routine and, if necessary, to refrain from outdoor activities in extreme heat or to postpone them until the early morning hours.

Some people with atopic dermatitis also suffer from a pollen allergy, which further aggravates the symptoms during heat, sun, and pollen exposure [76]. The altered pollen flight due to climate change can thus lead to increased symptom severity for this group [77].

Certain diseases may occur together with atopic dermatitis as a result of exposure to harmful environmental factors or lack of protective factors. These include, in particular, allergies, allergic asthma, eosinophilic oesophagitis (allergy-like inflammation of the oesophagus), and urticaria. Atopic dermatitis is a major risk factor for the development of allergies [78]. These diseases are thought to be linked through complex genetic, epigenetic, and immunological mechanisms.

From demographic change and the increasing incidence of atopic diseases in middle and advanced age, it follows that older persons and those with comorbidities are also increasingly becoming vulnerable groups to the consequences of climate change.

### 2.4 Air pollutants: Influence of air pollution on pollen grains, aeroallergens and allergic reactions

Experimental studies show that the combined effect of pollen and air pollutants is particularly unfavourable for people with allergies. The effects of air pollutants on health, as described by Breitner-Busch et al. [79] in this status report, and on respiratory allergies in particular, depend on a combination of factors including concentrations of environmental pollutants (e.g. nitrogen dioxide (NO_2_), carbon monoxide (CO), ozone (O_3_)), duration of exposure, ventilation, climatic conditions, and the interaction between pollutants and pollen. Air pollution seems to have several effects on pollen grains: changes in biological and reproductive functions, alterations of physico-chemical properties of the pollen surface, alterations of allergenic potential, and an adjuvant effect that increases potential health risks [80].

#### Pollen wall damage, allergen release and distribution in the environment

The flowering and pollen seasons are sensitive to environmental variability in terms of meteorological parameters, but also in terms of pollutants. Recently, an earlier flowering season has been demonstrated due to the combination of higher temperatures and degree of urbanisation. This is observed most clearly at sites with higher NO_2_ concentrations [40]. Additionally, a decrease in viability and/or germination of pollen exposed to very low O_3_ or NO_2_ concentrations in vitro has been observed in several species [81, 82]. It can be assumed that pollen in nature is exposed to both pollutants simultaneously and thus synergistic effects can be expected, since the presence of O_3_ increases NO_2_ uptake. This favours the nitration of proteins and thus impairs protein/enzyme functions [83].

According to various studies, pollen grains in areas with high air pollution are smaller and more fragile than in areas with lower air pollution. The interaction between air pollutants and pollen grains could damage the pollen wall and increase the number of allergens released into the environment [84], which eventually penetrate the lower airways and cause asthma-related symptoms.

There is some evidence that climate change and air pollutants as plant stressors change the morphology of antigens and thus the allergenic potential of pollen particles. Pollen from urban areas and from more polluted regions has a higher allergen content per pollen grain [85]. A higher allergen content was detected in extracts from birch pollen exposed to high O_3_ concentrations [86].

In addition, some studies show that allergenicity and viability of some pollen species increase when vegetation is increasingly polluted with certain pollutants. NO_2_, a major traffic-related air pollutant, was found to increase the allergenicity of the birch pollen allergen Bet v 1 [87].

Finally, several air pollutants act as adjuvants (amplifying agents) by binding to allergens and stimulating IgE synthesis, leading to an exacerbation of asthma symptoms. Several in vitro studies have shown that traffic-related air pollutants can modify pollen, increasing the frequency and intensity of symptoms in allergic individuals [80, 88]. In predisposed individuals, airway sensitisation to aeroallergens may be promoted [89]. By inducing airway inflammation, pollutants can damage the mucosal barrier, triggering an allergenic response [90]. Damage to the airway mucosa can facilitate the access of inhaled allergens to the cells of the immune system.

## 3. Recommendations

### 3.1 Pollen and fungal spore monitoring

#### Clinical and societal significance of pollen and fungal spore monitoring

There are various pharmacological and non-pharmacological measures for the prevention and treatment of allergic respiratory diseases caused by pollen or fungal spores [91]. One of the primarily non-pharmacological approaches is to be able to inform oneself – as the person affected or as the attending physician – about when, where, and in what quantity allergy-triggering pollen or fungal spores are present in the air in order to (a) avoid these places if possible and (b) be able to take medication before the onset of symptoms if necessary.

Continuous pollen monitoring also makes it possible to observe local, regional or transnational changes in the pollen spectrum and pollen flight. For such observations, which can be used as one of the tools in uncovering the relationships between climate change, nature, and health, it is necessary to ensure high-quality and long-term measurements of a broad spectrum of pollen and fungal spores at the same sites, as provided by the PID since 1983.

#### Air sampling and analysis of pollen and fungal spores

Air sampling can be passive, by sedimentation or filtering of the air, or active, by aspiration of a defined volume of air using a volumetric pollen and spore trap. The subsequent analysis of the airborne dust samples for pollen and fungal spores is mainly carried out by pollen analysts using light microscopy, less frequently by DNA analysis or by newly developed automated identification of pollen taxa using various methods (e.g. digital microscopy, fluorescence) [92–96].

#### Status quo of pollen and fungal spore monitoring in Germany

**Figure 4:**
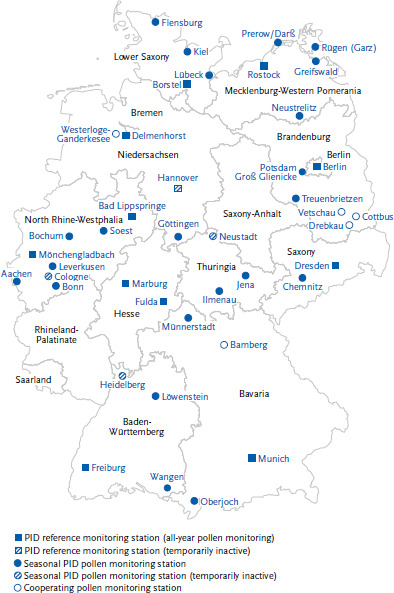
Pollen and spore measuring stations in the PID monitoring network as of January 2023 Source: German Pollen Information Service Foundation [97] (original language German)

As in most European countries, there is a nationwide pollen monitoring network in Germany, which has been operated by the PID since 1983. The sites of the current monitoring stations are shown in Figure 4.

The PID monitoring network currently operates on the basis of the Hirst-type volumetric spore trap [93] and light microscopic pollen analysis [95]. Part of the measured pollen data is used for the pollen forecast (Pollenflug-Gefahrenindex) for eight allergy-relevant pollen types provided by the Deutscher Wetterdienst (DWD; German Meteorological Service) [98]. As pollen from other plants and fungal spores also trigger sensitisation and allergies, monitoring by the PID covers a wider spectrum of pollen types as well as some allergy-relevant fungal spores, specifically *Alternaria*, *Cladosporium*, *Epicoccum*, and *Pleospora* at a limited number of monitoring stations. Based on these data, the PID publishes detailed weekly pollen and spore forecasts for Germany [99]. The nationwide pollen monitoring also forms the basis for national and regional pollen calendars, which are reissued every few years [25].

#### Further development of pollen monitoring

The long-term maintenance and further development of nationwide pollen monitoring as well as the further development and expansion of fungal spore monitoring can only be guaranteed if funding is secured.

To this end, the interdisciplinary Working Group Nationwide Pollen Monitoring was formed in 2017 and developed a position paper on perspectives for nationwide pollen monitoring in Germany [100]. The paper addresses: (a) the health and economic importance of pollen and pollen data; (b) methods for measuring pollen data; (c) the status quo of the nationwide pollen monitoring network in Germany; (d) pollen monitoring networks in other European countries; (e) legal framework conditions in Germany; and (f) possibilities for a reliable nationwide pollen monitoring network. In its conclusion, the working group provides the following recommendation:

‘Due to the importance of allergenic pollen to human health and allergic diseases for the healthcare system, the working group is in favour of including nationwide pollen monitoring in the catalogue of governmental responsibilities that provide the basic supply of the population with essential goods and services (services of general interest)’. It further states: ‘With regard to possible responsibilities within the framework of services of general interest, several approaches were discussed in the working group. These included the possibility of entrusting a federal institution, such as the DWD, with the continuation and further development of the nationwide pollen monitoring network. Another possibility would be to transfer the task to the German Pollen Information Service Foundation or other institutions. Regardless of the future responsibility, the cooperation of metrological, clinical, and scientific institutions is of fundamental importance for adequate preventive health measures’ ([100, P. 659] original language German).

### 3.2 Allergy and sensitisation monitoring

#### Monitoring at population level

Allergies are a highly relevant public health issue due to the large number of people affected in Germany. In order to address the problem of allergies affected by climate change by suitable primary, secondary, and tertiary prevention measures, the description of the current state and the observation of developments over time (trends) need to be continuously provided by indicator-based surveillance. Surveillance is defined by the World Health Organization as ‘the systematic on-going collection, collation and analysis of data […] and the timely dissemination of public health information for assessment and public health response’ [101, P. 14].

Suitable allergy indicators can be found, on the one hand, in the frequency and therapy of manifest diseases with clinical symptoms. On the other hand, sensitisation is relevant as a disease-related risk factor, since the immune system classifies an initially harmless environmental substance (allergen) as harmful and reacts with an allergen-specific immune response. After sensitisation, any further contact with the allergen can lead to symptoms. In Germany, existing health monitoring at the Robert Koch Institute with population-representative interview and examination surveys of adults (DEGS) and children/adolescents (KiGGS) provides a sound basis for the establishment of public health surveillance that includes allergies as non-communicable diseases [102]. Data should come from surveys and examinations as well as from official statistics and billing and care data. In addition, the courses of the most common allergic diseases (including the type I diseases, allergic rhinitis and allergic bronchial asthma, and the type IV disease, allergic contact dermatitis) should be investigated jointly across several age groups.

#### Monitoring at patient level

In addition to continuous monitoring at the population level, patient-related monitoring systems can efficiently record the diagnosis, severity, and course of allergic diseases within the healthcare system. The underlying data are usually collected at very close intervals on a centre- or study-specific basis [60, 61].

For adequate care of patients, especially those with inhalation allergies, such a system at the patient level makes sense as a supplement to sensitisation monitoring at the population level. In addition to a registry, sensitisation monitoring at patient level could specifically include allergens that could become clinically relevant but currently play no or only a minor role in everyday clinical practice, such as Amb a 1, the main allergen of ragweed. This requires suitable diagnostic tools to be available for rare and new allergens [61].

The concrete design of such a monitoring system could be developed in an interdisciplinary manner, analogous to the perspectives for a nationwide pollen monitoring system developed by the Working Group Nationwide Pollen Monitoring [100].

### 3.3 Urban planning from an allergological perspective

Urban green spaces can reduce some of the negative impacts of climate change. Parks, road-side trees, green facades, and roofs create recreational spaces, while cold islands and cool buildings provide shade, improve air quality, and have a positive effect on people’s overall well-being [15].

However, which urban greenery should be planted? This question can be approached from different points of view. For example, the German Conference of Garden Authorities (Deutsche Gartenamtsleiterkonferenz, GALK) has selected ‘future trees for the city’ from its list of road-side trees in 2022, which has been maintained and regularly revised since the 1970s and takes the aspect of climate robustness into account [103].

From an allergological point of view, the aspect of the allergenic potential of urban green spaces must also be considered (e.g. [104]). It would not make sense from either a health or an economic perspective to continue planting trees to whose pollen a large number of people in Germany are allergic [105] or could develop an allergy during their lifetime [106]. It would also be ill-advised to resort to previously non-native trees that are adapted to high air temperatures but have a high allergenic potential, such as the olive tree [60]. In order to address the allergological aspect of urban green spaces, Bergmann et al. [107] recommended in 2012 that cities and municipalities should be considerate of pollen allergy sufferers when planting public spaces. They published a list of tree and shrub species resident in Berlin that are suitable for new plantings from an allergological perspective. This list is currently being revised and should be merged with the GALK list of future trees.

### 3.4 Responding to changes in the working environment

**Figure 5:**
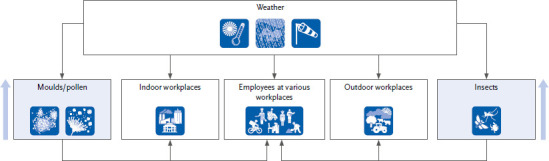
Influence of climate change factors on the environment and thus on employees in different workplaces. Only a few allergen sources for which an increase in exposure is to be expected are shown as examples. Source: Own representation based on Raulf, Hut (Institute for Prevention und Occupational Medicine)

Although a precise assessment of the health and economic consequences of climate change on the working environment is currently not possible, the risks to occupational safety and health associated with climate change must be increasingly addressed in global occupational legislation (Figure 5). Adapted assessment scales and protective measures need to be provided. This was done, for example, for the effects of increased UV radiation exposure during outdoor employment. Certain UV-related skin cancers were introduced in the official occupational disease list and are now recognised occupational diseases in Germany (BK 5103) [108]. In addition to climate change-related stressors such as heat and UV radiation, another focus should lie on infectious diseases and allergies.

It is also important to adequately inform affected occupational groups about the possible effects of climate change and to implement targeted preventive measures. Prevention, especially of allergic diseases, includes early and targeted diagnostics that are continuously adapted to the changing conditions. Research is required to expand knowledge regarding the type, distribution, and impact of allergens, for example, which can serve as a basis for preventive measures. Climate change reaches all areas of society and does not stop at occupational health and safety.

## 4. Summary and outlook

Inhalation allergies in particular, amongst other atopic diseases, have increased worldwide and reached epidemic levels. Climatic changes influence flora and fauna and therefore the occurrence of aeroallergens. Polysensitised pollen allergic patients can suffer from allergy symptoms almost all year round due to changes in the flowering period of plants. More detailed information on climate change and (new) pollen allergens can be found in Section 2.1 Climate change and (new) pollen allergens.

The triggering of allergic symptoms is always preceded by the phase of sensitisation through allergen exposure. Environmental factors such as air pollutants and climate influence the allergenicity of pollen through chemical modifications and accumulation of allergens (agglomeration), which can lead to the formation of new allergens (neoallergens) [109, 110]. Environmental pollutants can promote the penetration of allergens into the skin and mucous membranes by damaging the skin and mucous membrane barrier, modulating the immune system, causing inflammation and thereby influencing individual susceptibility to developing allergies [109]. More detailed information on these topics can be found in Section 2.3 Atopic dermatitis and climate change and Section 2.4 Air pollutants.

Symptoms of allergic reactions are triggered by released mediators. The strength of the reaction depends on the allergen concentration and thus, on the one hand, on the pollen concentration and, on the other hand, on the quantity and structure of the allergens released from pollen, which in turn also depend on climatic conditions [49]. This becomes clear in a phenomenon that has gained importance, known as thunderstorm asthma. People with hay fever can suffer severe asthma attacks in such exceptional situations, which they had not experienced before [111]. A more detailed insight into the phenomenon of thunderstorm asthma is given in the Info box above.

Storms and heavy rain lead to flooding; damp flats are predestined for mould and bacterial growth. People who occupy damp apartments or houses are at risk, as well as workers involved in renovation and demolition [112, 113]. Climatic changes lead to changes in flora, fauna, and funga, i.e. the fungi found in an area. Increasing temperatures favour the growth of plants and fungi as well as the spread of animals, including pests, which are otherwise native to warmer regions. Insect components are considered relevant aeroallergens, especially in warm climates. More detailed information can be found in Section 2.2 Climate change and (new) occupational inhalant allergens.

For targeted allergological diagnostics, all allergens must be available, even those that have not been of high relevance in Germany so far. Currently, allergen immunotherapy is still the only treatment option that aims to induce tolerance to individual allergens in patients. Allergen extracts may have to be adapted to the respective sensitisation profile. Since climatic changes can lead to plants expressing new allergens or to changes in the quantitative composition of allergens; it must be ensured that the relevant allergens are contained in adequate concentrations in extracts. More detailed explanations on the topic of sensitisation monitoring and further recommendations can be found in Section 3 Recommendations.

Climate change is increasingly influencing our health and our lives. Politicians and society, and especially healthcare workers, are called upon to consider findings from basic scientific research, environmental, working environment and disease monitoring in their actions.

## Key statements

- Allergic diseases, especially inhalation allergies, have reached epidemic levels. One of the most common triggers of inhalation allergies are the allergens contained in pollen.
- The flowering and therefore the timing of pollen occurrence is changing. Changes in pollen concentration and in the spectrum of allergenic pollen are also to be expected.
- Outdoor workers in particular are affected by exposures to plant and animal profiteers of climate change.
- Pollen monitoring is an important tool for uncovering the relationships between climate change and health and should be included in services of general interest.
- Allergy and sensitisation monitoring should be permanently established at both population and patient levels.
- The allergenic potential of plants must be considered when planning urban green spaces.
- The risks to occupational health and safety associated with climate change must be increasingly addressed in global occupational health and safety measures.

## Figures and Tables

**Table 1:** Overview of allergic reaction types Source: Allergieinformationsdienst [1] (original language German)

Name (type)	Reaction process	Duration from contact to reaction	Examples
Type I, immediate-type	Mediation through allergen-specific IgE-antibodies, release of mediators (especially histamine)	Seconds to minutes (potential late-phase reaction after 4–6 hours)	Allergic rhinitis/conjunctivitis, allergic asthma, urticaria, insect venom allergy, anaphylaxis
Type II, cytotoxic type	Formation of antigen-antibody-complexes, destruction of the body’s own cells	6–12 hours	Transfusion reactions, some drug reactions and autoimmune diseases
Type III, immune complex type	Formation of antigen-antibody-complexes, release of tissue-damaging substances	6–12 hours	Allergic vasculitis, serum sickness, exogenous allergic alveolitis (e.g. farmer’s lung)
Type IV, contact/delayed-type	Mediation through cells (allergen-specific T lymphocytes)	12–72 hours	Allergic contact dermatitis, drug reactions, transplant rejection reaction

**Table 2:** Annual pollen sums of ragweed in 2010 at monitoring sites in North Rhine-Westphalia (NW), Bavaria, and Brandenburg Source: Höflich [56] (original language German)

Location of monitoring station	Annual pollen sum (pollen/m^3^)
North Rhine-Westphalia (Mönchengladbach)	5
Bavaria (Munich)	6
Brandenburg (Drebkau)	Nearly 2,500

Data from one monitoring station each. Data source NW, Bavaria: German Pollen Information Service Foundation, for details see Höflich et al. [50]. Data source Brandenburg: Ministry of Environment, Health and Consumer Protection Brandenburg [57]
